# Fracture Risk in Dialysis and Kidney Transplanted Patients: A Systematic Review

**DOI:** 10.1002/jbm4.10067

**Published:** 2018-07-05

**Authors:** Aboubacar Sidibé, David Auguste, Louis‐Charles Desbiens, Catherine Fortier, Yue Pei Wang, Sonia Jean, Lynne Moore, Fabrice Mac‐Way

**Affiliations:** ^1^ Centre de Recherche du CHU de Québec Hôpital Hôtel‐Dieu de Québec Division of Nephrology, Endocrinology, and Nephrology Axis Faculty of Medicine Department of Social and Preventive Medicine Laval University Quebec Canada; ^2^ Centre de Recherche du CHU de Québec Hôpital Saint‐Sacrement Faculty of Medicine Department of Social and Preventive Medicine Laval University Quebec Canada; ^3^ Centre de Recherche du CHU de Québec Hôpital Hôtel‐Dieu de Québec Division of Nephrology, Endocrinology, and Nephrology Axis Faculty and Department of Medicine Laval University Quebec Canada; ^4^ Institut National de Santé Publique du Québec Medicine Faculty Department of Social and Preventive Medicine Laval University Quebec Canada; ^5^ Centre de Recherche du CHU de Québec Hôpital de l'Enfant‐Jésus Traumatology Axis Medicine Faculty Department of Social and Preventive Medicine Laval University Quebec Canada

**Keywords:** CKD‐MBD, FRACTURES, HEMODIALYSIS, PERITONEAL DIALYSIS, KIDNEY TRANSPLANTATION

## Abstract

Chronic kidney disease is associated with an increased risk of fracture and cardiovascular mortality. The risk of fracture in hemodialysis (HD), peritoneal dialysis (PD) and kidney transplant (KT) patients is higher when compared with the general population. However, there exists a knowledge gap concerning which group has the highest risk of fracture. We aimed to compare the risk of fracture in HD, PD, and KT populations. We conducted a systematic review of observational studies evaluating the risk of fracture in HD, PD, or KT patients. Eligible studies were searched using MEDLINE, Embase, Web of Science, and Cochrane Library from their inception to January 2016, and in grey literature. Incidences (cumulative and rate) of fracture were described together using the median, according to fracture sites, the data source (administrative database or cohort and clinical registry), and fracture diagnosis method. Prevalence estimates were described separately. We included 47 studies evaluating the risk of fracture in HD, PD, and KT populations. In administrative database studies, incidence of hip fracture in HD (median 11.45 per 1000 person‐years [p‐y]), range: 9.3 to 13.6 was higher than in KT (median 2.6 per 1000 p‐y; range 1.5 to 3.8) or in PD (median 5.2 per 1000 p‐y; range 4.1 to 6.3). In dialysis (HD+PD), three studies reported a higher incidence of hip fracture than in KT. Prevalent vertebral fracture (assessed by X‐rays or questionnaire) reported in HD was in a similar range as that reported in KT. Incidence of overall fracture was similar in HD and KT, from administrative databases studies, but lower in HD compared with KT, from cohorts or clinical registry studies. This systematic review suggests an important difference in fracture risk between HD, PD, and KT population, which vary according to the diagnosis method for fracture identification. © 2018 The Authors. *JBMR Plus* published by Wiley Periodicals, Inc. on behalf of American Society for Bone and Mineral Research.

## Introduction

Chronic kidney disease (CKD) is a major public health issue worldwide. In 2011, more than 615,000 people suffered from end‐stage renal disease (ESRD) in United States.^1^, ^2^ In 2012, the unadjusted prevalence of ESRD was 716.7 per million person (pmp) in Europe,^3^ whereas 35,281 Canadians (excluding the province of Quebec) were suffering from ESRD in 2014.^4^ Loss of kidney function leads to metabolic disorders that affect bone and vascular health known as CKD‐mineral and bone disorder (CKD‐MBD).^5^, ^6^, ^7^ Clinically, CKD‐MBD is associated with an increased risk of fracture and cardiovascular mortality.^8^, ^9^, ^10^, ^11^, ^12^ Patients with ESRD will eventually require a renal replacement therapy (RRT) and will therefore be treated by hemodialysis (HD), peritoneal dialysis (PD), or kidney transplantation (KT).^13^ The increased risk of fracture in HD, PD, and KT patients compared with the general population has been recognized.^8^, ^14^, ^15^ Indeed, hip fracture has been shown to be the most common type of fracture in ESRD with a fracture rate 17.2 times greater than that observed in the general population.^8^, ^15^ This association was also reported in age, sex, and race subgroups.^8^ The risk of vertebral fracture is also higher in older women with decreased kidney function.^16^ However, there is currently a knowledge gap on whether the risk of fracture differs between the HD, PD, and KT population.

Whereas Beaubrun and colleagues^17^ reported in the United States that the incidence rate of hip fracture in HD patients was 20.6 per 1000 persons‐years in 2009, Nair and colleagues^18^ reported a much lower incidence rate of 3.8 per 1000 person‐years in KT patients. In contrast, another study reported that the risk of hip fracture in the first 3 years post‐KT was 1.34‐fold that of dialysis patients,^19^ which is mainly explained by the use of high corticosteroids to prevent graft rejection. After the first 3 years post‐KT, the risk of fracture declined and tended to be equal that of HD patients.^19^ When comparing patients in dialysis, a recent study^2^ showed that the risk of hip fracture in HD was 1.74‐fold that in PD, whereas another study did not find any difference between HD, PD, and KT patients.^9^ Given these disparities, we conducted a systematic review to identify the risk of fracture and cardiovascular mortality post‐fracture in HD compared with PD or KT and in PD compared with KT populations.

## Materials and Methods

### Study design

Based on a protocol registered on Prospero (CRD42016037526) that was recently published,^20^ we conducted a systematic review following the methodological recommendations of the Cochrane Handbook for Systematic Reviews of Interventions^21^ and reported the results using the Preferred Reporting Items for Systematic Reviews and Meta‐Analyses (PRISMA) statement.^22^

### Eligibility criteria

We included observational studies (cohort studies, cross‐sectional studies, case‐control studies) conducted in CKD adults ≥18 years (at least 80% of participants) treated by either HD, KT, or PD and evaluating the risk of fracture (hip, vertebral, and/or overall fracture) without a comparator or compared with a renal replacement therapy (HD, KT, PD), non‐dialyzed CKD, or general population. The primary outcome was the risk (incidence rate, incidence proportion, odds or prevalence) of fracture. Secondary outcomes were fracture sites (hip, vertebral, overall fracture), risk of cardiovascular mortality post fracture, all‐cause mortality associated with fracture, length of hospitalization post fracture and number of hospitalizations post fracture (during the following years).

### Information sources and search strategy

We performed a search using electronic databases (Medline, Cochrane Library, Embase, and Web of Science), from their inception until January 2016. Our search strategy was based on key words related to the intervention (KT, HD, PD) and the outcome (fracture). A search strategy was first elaborated for Pubmed/Medline and Embase (Supplemental Table S1) then adapted to Cochrane Library and Web of Science with no restriction of language or year of publication. We then hand‐searched reference lists of relevant articles and the Grey literature (Google Scholar, thesis repositories including Thesis portal Canada, EtHOS, DART‐Europe E‐Thesis Portal, the National Library of Australia's Trove, and ProQuest Dissertations & Theses Global).

### Study selection and data management

After removing duplicates of identified records from our search strategies using EndNote (version X7.2.1, Thomson Reuters, New York, NY, USA, 1988–2014), two independent reviewers screened each study by title and abstract using standardized and pilot tested screening forms. Full texts were also screened when titles and abstract were insufficient to establish inclusion of a study in the review.

### Data extraction and risk of bias assessment

Data of included studies were then independently extracted, using a standardized and piloted tested data extraction form. In each step, discrepancies between the two reviewers (AS and CF) were resolved through consensus or with the involvement of a third reviewer (FM), as required. Extracted data included information on the study, characteristics of the study population and intervention (HD, PD or KT), comparator, and outcomes. Primary investigators of included studies were contacted when needed. Risk of bias was assessed with a tool developed by the Cochrane Collaboration (ROBINS‐I tool).^23^ Risk of bias was judged as low (the study is comparable to a well‐performed randomized trial), moderate (the study provides sound evidence for a non‐randomized study but cannot be considered comparable to a well‐performed randomized trial), serious (the study has some important problems), critical (the study is too problematic to provide any useful evidence and should not be included in any synthesis), or no information (no information on which to base a judgment about risk of bias). Information on the source of funding was collected for each study to assess conflicts of interest.

### Data analysis

Frequencies of fracture in included studies were first described separately as reported, then characterized using the median and interquartile range as summary measures for each RRT group (HD, PD, KT, or combined dialysis [HD + PD]) according to the fracture site (hip, vertebral, or overall fracture), the data source (administrative database or cohort and clinical registry), and the fracture diagnosis method. Prevalence of fracture is reported separately, while cumulative incidence was converted to incidence rate using the statistical approach recommended by Rothman.^24^ For studies assessing the association between RRT and fracture and where a measure of association was available, we reported these measures by intervention group‐comparator and fracture's site.

## Results

**Figure 1:**
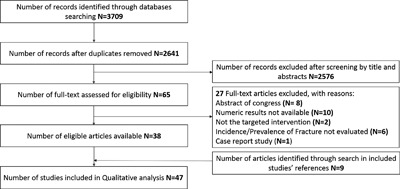
Study selection's flow diagram. This figure describes the study selection process.

We identified 2641 references from electronic and hand searches, and included 47 studies that evaluated the risk of fracture in HD, PD, and/or KT patients (Fig. 1), with sample sizes ranging from 29 to 935,621. Characteristics of the included studies are described in Tables 1, 2, and 3. Among the included studies, 22 were conducted in the United States, 13 in Europe, 10 in Asia, and 2 were multinational. The mean follow‐up ranged from 1 to 10 years. We found 18 studies that evaluated the risk of fracture in KT group, without a comparator in 14 studies,^18^, ^24^, ^25^, ^26^, ^27^, ^28^, ^29^, ^30^, ^31^, ^32^, ^33^, ^34^, ^35^, ^36^ compared with the general population in 3 studies^37^, ^38^, ^39^ and compared with dialysis population in 1 study^19^ (Table 1). Concerning dialysis population (HD and PD), 5 studies reported the risk of fracture, without a comparator in 2 studies,^40^, ^41^ and compared with the general population in 3 studies.^8^, ^14^, ^42^ Only one study evaluated the risk of fracture in PD patients without a comparator^43^ (Table 2). In HD patients, 23 studies reported the risk of fracture, without a comparator in 15 studies,^44^, ^45^, ^46^, ^47^, ^48^, ^49^, ^50^, ^51^, ^52^, ^53^, ^54^, ^55^, ^56^, ^57^, ^58^ compared with the general population in 4 studies,^11^, ^59^, ^60^, ^61^ with PD in 3 studies,^2^, ^62^, ^63^ and with PD and KT in 1 study^9^ (Table 3).

**Table 1:** Characteristics of Studies Evaluating the Risk of Fracture in Kidney Transplant According to the Design and Comparator Group

First author, year of publication, and study country	Sample size	White (%)	Age, years (mean)	Female (%)	Mean follow‐up (years)	Mean follow‐up post fracture (years)	Hip fracture	Vertebral fracture	Overall fracture	Risk of bias
		Cross‐sectional study, kidney transplant versus no comparator								
Braga 2006, Brazil^24^	191	NR	44.8	50.8	NR	NA		√	√	Critical
Durieux 2002, France^25^	59	71.0	49.6	45.8	8.5	NA		√	√	Critical
Patel 2001, United Kingdom^26^	165	90.0	47.0	42.4	NR	NA		√		Critical
Nam 2000, Korea^27^	166	NR	40.0	34.3	NR	NA		√		Critical
Nisbeth 1999, Sweden^28^	193	NR	50.9	39.9	NR	NA		√	√	Critical
Grotz 1994, Germany^29^	100	NR	44.0	46.0	NR	NA			√	Critical
		Retrospective cohort study, kidney transplant versus no comparator								
Ferro 2015, United Kingdom^30^	21,769	71.1	NR	38.7	5.7	NR	√		√	Critical
Nair 2014, United States^18^	69,740	56.9	50.5	39.0	2.2	1^b^	√			Moderate
Nikkel 2012, United States^31^	77,430	65.8	48.8	39.7	3.9	NA			√	Critical
Opelz 2011, multinational^32^	20,509	86.9	47.9	38.4	5.0b	NA	√			Critical
Nikkel 2009, United States^33^	68,814	73.8	43.7	39.7	5.0b	NR			√	Critical
O'Shaughnessy 2002, United States^34^	1572	NR	NR	41.2	6.5	NR	√	√	√	Critical
		Prospective cohort study, kidney transplant versus no comparator								
Ramsey‐Goldman 1999, United States^35^	432	54.0	41.3	40.0	2.1	NA			√	Critical
Elmstedt 1981, Sweden^36^	204	NR	NR	42.7	6.2	NA			√	Critical
		Retrospective cohort study, kidney transplant versus general population								
Naylor 2016,^a^ Canada^37^	4821	NR	49.3	36.9	2.9	NR	√		√	Moderate
Vautour 2004, United States^38^	86	92.0	38.3	31.4	10.6	NR		√	√	Serious
Abbot 2001, United States^39^	33,479	75.6	42.9	39.8	1.7	NR			√	Moderate
		Retrospective cohort study, kidney transplant versus dialysis								
Ball 2002, United States^19^	101,039	63.20	40.6	40.60	3.0	NR	√			Moderate

NR = Not Reported; NA = Not Applicable; √ = This type of fracture risk was assessed in the study.

^a^ Also compared fracture risk in KT to non‐dialysis CKD.

^b^ Total follow‐up time.

**Table 2:** Characteristics of Studies Evaluating the Risk of Fracture in Dialysis and Peritoneal Dialysis According to the Design and Comparator Group

First author, year of publication, and study country	Sample size	White (%)	Age, years (mean)	Female (%)	Mean follow‐up (years)	Mean follow‐up post fracture (years)		Hip FX	Vertebral FX	Overall FX	Risk of bias
		Retrospective cohort study, dialysis versus no comparator									
Nair 2013, United States^40^	409,040	76.5	76.0	48.1	NR	1^a^	√				Serious
Danese 2006, United States^41^	9007	53.8	61.7	42.5	NR	NR			√	√	Critical
		Retrospective cohort study, dialysis versus general population									
Maravic 2014, France^42^	29,487	NR	NR	40.0	1a	NR				√	Critical
Alem 2000, United States^14^	326,464	100.0	NR	44.1	NR	NR	√				Moderate
Coco 2000, United States^8^	1272	17.5	58.0	52.2	3.2	1^a^	√				Moderate
		Case‐control study, peritoneal dialysis versus no comparator									
Ma 2013, China^43^	24	NR	73.3	40.0	1.3	NR				√	Critical

FX = fracture; NR = Not Reported; √ = This type of fracture risk was assessed in the study.

^a^ Total follow‐up time.

**Table 3:** Characteristics of Studies Evaluating the Risk of Fracture in Hemodialysis According to the Design and Comparator Group

First author, year of publication, and country	Sample size	White (%)	Age, years (mean)	Female (%)	Mean follow‐up (years)	Mean follow‐up post fracture (years)	Risk of bias	Hip fracture	Vertebral fracture	Overall fracture
		Cross‐sectional study, hemodialysis versus no comparator								
Simunovic 2015, Croatia^44^	767	NR	NR	NR	NR	NA	NI			√
Fusaro 2013, Italy^45^	387	NR	64.2	37.0	NR	NA	Serious		√	
Mares 2009, Japan^46^	72	100.0	65.0	44.0	NR	NA	Critical		√	
Kaneko 2007, United States^47^	7159	50.4	58.4	48.2	3.3	NA	Critical			√
Inaba 2005, Japan^48^	114	100.0	73.1	100.0	NR	NA	Critical			
Urena 2003, France^49^	70	100.0	60.5	37.1	NR	NA	Critical		√	√
Fontaine 2000, Belgium^50^	88	NR	58.0	42.1	NR	NA	Critical		√	√
Atsumi 1999, Japan^51^	187	0	54.2	0	NR	NA	Critical		√	√
Mohini 1991, United States^52^	66	NR	NR	NR	NR	NA	Critical		√	
		Retrospective cohort study, hemodialysis versus no comparator								
Jamal 2006, Canada^53^	52	NR	66.0	28.85	NR	NR	Critical			√
Wagner 2014, United States^54^	935,221	NR	NR	NR	NR	NR	Critical			√
Chang 2013, Taiwan^55^	82,491	NR	NR	47.9	5.0^a^	NR	Critical			√
Wakasugi 2014, Japan^56^	128,141	NR	64.3	38.1	1.0^a^	NR	Serious	√		
Lavorato 2009, Brazil^57^	50	NR	NR	44.3	NR	NR	Serious	√		
		Prospective cohort studies, hemodialysis versus no comparator								
Jadoul 2006, multinational^58^	12,782	NR	NR	418	NR	NR	Serious	√		√
		Prospective cohort studies, hemodialysis versus general population								
Tentori 2014, multinational^59^	34,579	NR	65.0	41.1	1.6	0.6	Critical	√		√
Wakasugi 2013, Japan^60^	128,141	NR	64.3	38.1	1.0^a^	NR	Moderate	√		
Rodrıguez‐Garcıa 2009, Spain^11^	193	NR	65.5	37.3	2.0^a^	NA	Critical		√	√
Rodríguez‐García 2003, Spain^61^	99	NR	67.6	40.4	NR	NR	Serious		√	
		Retrospective cohort study, HD versus peritoneal dialysis								
Zhe‐Zhong 2014, Taiwan^62^	51,473	NR	60.4	52.1	4.1	NR	Moderate	√		
Mathew 2014, United States^63^	929,114	NR	NR	NR	NR	NR	Moderate	√		
Chen 2014, Taiwan^2^	64,124	NR	66.4	51.0	NR	NR	Moderate	√		
Stehman‐Breen 2000, United States^b^, 9	4952	52.1	59.7	48.3	2.9	NR	Moderate	√		

NR = Not Reported; NA = Not Applicable; NI = No Information; √ = This type of fracture risk was assessed in the study.

^a^ Total follow‐up time.

^b^ Also compared with kidney transplant.

### Hip fracture risk reported in HD, KT, PD, and dialysis (HD + PD) population

**Figure 2:**
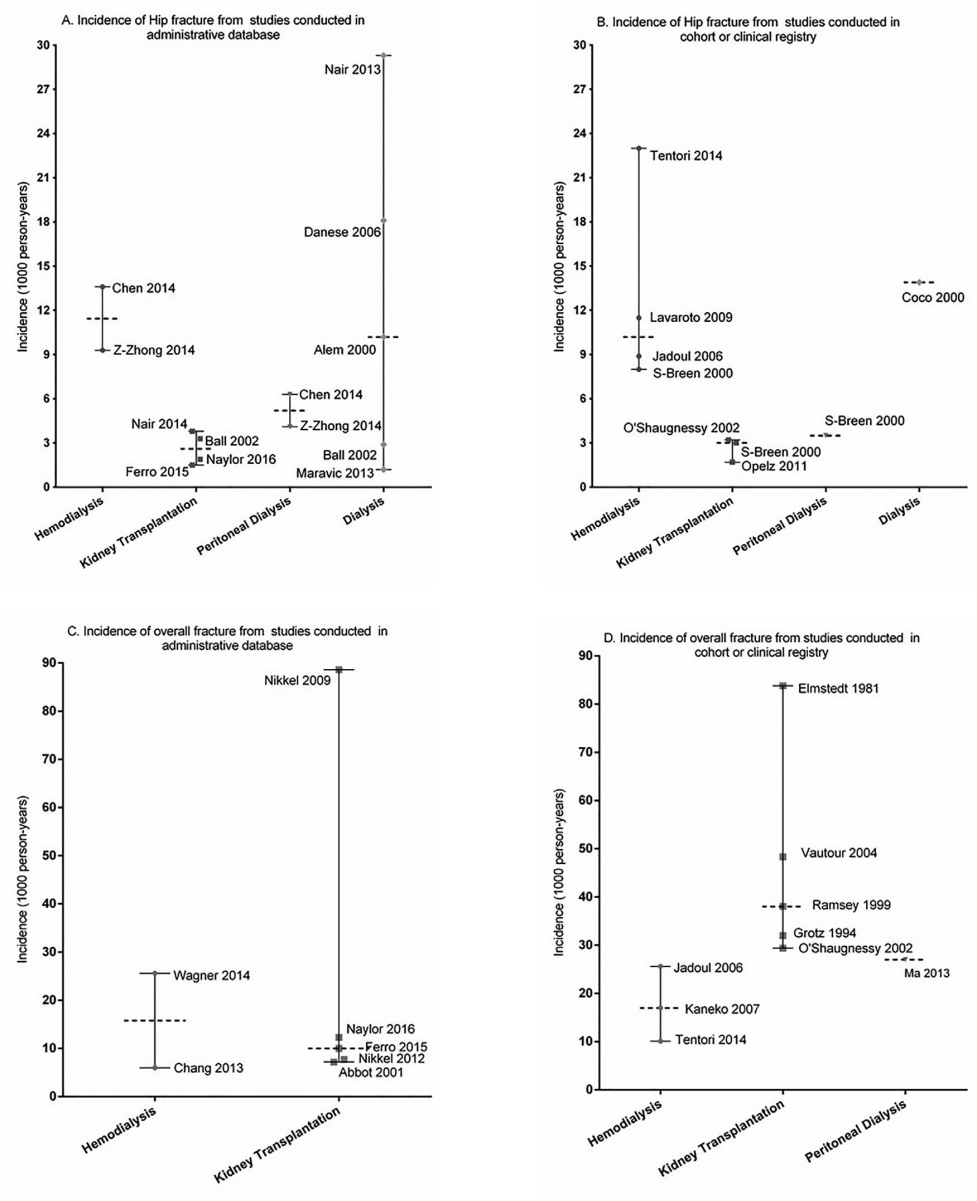
Incidence rates of hip fracture and overall fracture in dialysis and kidney transplant patients. (*A*, *B*) The incidence rate of hip fracture reported in studies is identified with a bullet and the first author's name and publication year according to the therapy group. The median incidence rate and range of hip fracture according to the therapy group are also presented. (*C*, *D*) Results are presented here for the incidence rate of overall fracture.

Incidence rate of hip fracture was reported by 10 studies^2^, ^14^, ^18^, ^19^, ^30^, ^37^, ^40^, ^41^, ^42^, ^62^ using administrative database and by 8 studies^9^, ^32^, ^34^, ^57^, ^58^, ^59^, ^60^ conducted in a cohort or a clinical registry. In administrative database studies, incidences of hip fracture reported by 2 studies in HD group^2^, ^62^ (median 11.45 per 1000 person‐years (p‐y); range 9.3 to 13.6) were higher than those reported by 4 studies^18^, ^19^, ^30^, ^37^ in KT group (median 2.6 per 1000 p‐y; range 1.5 to 3.8), or those reported by 2 studies^(2,62)^ 2 in PD group (median 5.2 per 1000 p‐y; range 4.1 to 6.3) (Fig. 2 *A*). In dialysis group (HD + PD), 3 of 5 studies^14^, ^19^, ^40^, ^41^, ^42^ reported an incidence rate of hip fracture (median 14.2 per 1000 p‐y; range 2.9 to 29.3) higher than that reported in the KT group (Fig. 2 *A*). Only one study^28^ reported a prevalence of hip fracture in a KT group (4.2%). The results were similar in studies conducted with cohorts or clinical registries. Indeed, incidences of hip fracture reported by 4 studies in HD group^9^, ^57^, ^58^, ^59^ were higher than those reported by 3 studies^9^, ^32^, ^34^ in KT group (Fig. 2 *B*). Only 1 study reported an incidence of hip fracture in a PD^9^ or dialysis group,^8^ estimated respectively at 3.5 and 13.9 per 1000 p‐y.

### Vertebral fracture risk in dialysis and kidney transplant population

**Figure 3:**
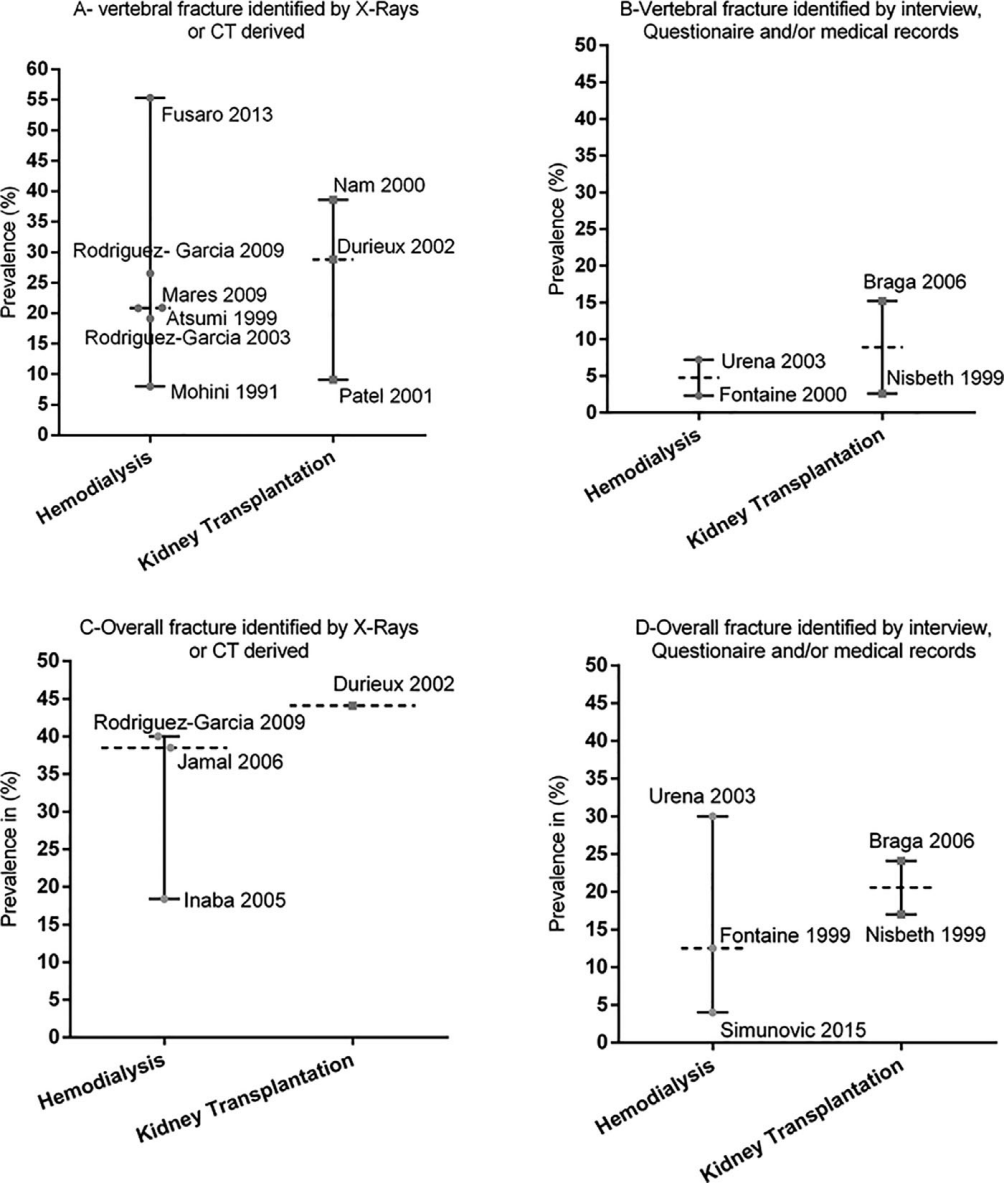
Prevalence of vertebral and overall fracture in hemodialysis and kidney transplant patients. (*A*, *B*) The prevalence of vertebral fracture in studies is identified with a bullet and the first author's name and publication year according to the therapy group. The median prevalence and range of vertebral fracture according to the therapy group are also presented. (*C*, *D*) Results are presented here for the prevalence of overall fracture.

Two studies^34^, ^38^ reported the incidence of vertebral fracture in KT group (7.2 and 15.4 per 1000 p‐y), whereas only 1 study^41^ reported this incidence in dialysis group (4.8 per 1000 p‐y). Incident vertebral fracture was assessed by clinical history and X‐rays,^38^ using outside medical records and phone contact^34^ and/or by inpatients claims.^41^ Prevalent vertebral fracture was assessed by X‐rays in 7 studies,^11^, ^25^, ^26^, ^27^, ^45^, ^51^, ^61^ interview or medical records in 2 studies,^49^, ^50^ CT‐scan in 1 study,^46^ and interview alone in 2 studies.^24^, ^28^ In 6 HD group studies that used X‐rays to assess vertebral fracture,^11^, ^45^, ^46^, ^51^, ^52^, ^61^ the prevalence was similar to that reported in 3 KT group studies^25^, ^26^, ^27^ (Fig. 3 *A*). No study reported vertebral fracture risk in PD patients. The results were also similar in studies that assessed vertebral fracture using interview, questionnaire, and/or medical records^24^, ^28^, ^49^, ^50^ (Fig. 3 *B*). The prevalence of vertebral fracture reported in dialysis and KT populations are further detailed in Supplemental Table S2.

### Overall fracture risk in dialysis and kidney transplant populations

Seven studies reported an incidence rate of overall fracture from an administrative database^30^, ^31^, ^33^, ^37^, ^39^, ^54^, ^55^ compared with 9 studies^29^, ^34^, ^35^, ^36^, ^38^, ^43^, ^47^, ^58^, ^59^ from a cohort or a clinical registry. From administrative databases, the incidences of overall fracture reported in 2 HD group studies^54^, ^55^ were similar to those reported in 5 KT group studies^30^, ^31^, ^37^, ^39^, ^64^ (Fig. 2 *C*). From cohorts or clinical registries, the incidences of overall fracture reported in 3 HD group studies^47^, ^58^, ^59^ (median 17.0 per 1000 p‐y; range 10.1 to 25.6) were lower than those reported by 5 KT group studies^29^, ^34^, ^35^, ^36^, ^38^ (median 38.0 per 1000 p‐y; range 29.4 to 83.8). Only 1 study^43^ reported an incidence of overall fracture using an administrative database in a PD group without any comparative study (Fig. 2 *D*). In studies that used X‐rays to diagnose fracture, 3^11^, ^48^, ^53^ reported the prevalence of overall fracture (median 12.5 per 1000 p‐y; range 4.0 to 30.0), whereas only 1 study^25^ reported that prevalence in a KT group (Fig. 3 *C*). In studies that used interview, questionnaire, and/or medical records to assess fracture, the prevalence of overall fracture reported in 3 HD group studies^44^, ^49^, ^50^ was similar to that reported by 2 KT group studies^24^, ^28^ (Fig. 3 *D*).

### Comparison of fracture risk in HD, PD, and KT groups versus non‐dialyzed CKD or general population

Three studies^37^, ^38^, ^39^ reported a higher risk of overall fracture in KT patients compared with the general population. Three other studies^8^, ^14^, ^42^ observed a higher risk of hip fracture in dialysis compared with the general population (Supplemental Table S3). In HD, 2 studies^59^, ^60^ reported a higher incidence of hip fracture compared with general population, whereas Rodriguez‐Garcia and colleagues^11^, ^61^ did not observe a significant difference between the prevalence of vertebral fracture in HD and the general population, but no measure of association was provided (Supplemental Table S3).

### Comparison of fracture risk in HD versus PD versus KT or non‐dialyzed CKD

One study^19^ reported a higher risk of hip fracture in KT patients compared with dialysis patients, whereas another study^9^ did not observe a difference in hip fracture risk when comparing HD with PD and KT patients. Three studies^2^, ^62^, ^63^ reported a significantly higher risk of hip fracture in HD versus PD patients. Finally, Naylor and colleagues^37^ recently observed a higher risk of overall fracture in KT patients compared with non‐dialyzed CKD (Supplemental Fig. S1).

### Mortality post fracture

Five studies^8^, ^40^, ^41^, ^42^, ^62^ evaluated the risk of overall mortality post fracture in dialysis population. Three of these studies reported a higher mortality rate in fractured dialysis patients compared with the general population,^8^ non‐dialysis,^42^ or non‐fractured dialysis population randomly selected.^41^ Mortality within 30 days post hip fracture in dialysis patients ≥67 years was 17.40% (95% confidence interval [CI] 16.9% to 18.0%) in the study from Nair and colleagues.^40^ Likewise, Zhe‐Zhong and colleagues^62^ reported a mortality rate of 3.2% in dialysis patients after hip fracture. When considering only patients in HD, Rodriguez‐Garcia and colleagues,^11^ Kaneko and colleagues,^47^ and Tentori and colleagues^59^ reported a higher mortality rate post fracture in HD compared with the general population, which exceeded 500 per 1000 p‐y in the later study. In KT population, the 30‐day mortality rate post‐fracture was 2.2 per 100 events as reported by Nair and colleagues^18^ and 20.7 per 100 events as reported by Ferro and colleagues^30^ (Supplemental Table S4).

### Hospitalization stays and cost post‐fracture

Only 1 study^42^ evaluated this outcome and reported a longer length of hospitalization stays and higher hospitalization costs due to fracture in dialysis versus non‐dialysis population (Supplemental Table S4).

### Risk of bias

In studies evaluating the incidence or prevalence of hip, vertebral, or overall fracture in HD, PD, KT, or dialysis, the majority were at critical risk of bias when evaluating fracture.^18^, ^24^, ^25^, ^26^, ^27^, ^28^, ^29^, ^30^, ^31^, ^32^, ^33^, ^34^, ^35^, ^36^, ^40^, ^41^, ^43^, ^44^, ^45^, ^46^, ^47^, ^48^, ^49^, ^50^, ^51^, ^52^, ^53^, ^54^, ^55^, ^57^, ^58^, ^60^ Five studies^40^, ^45^, ^56^, ^57^, ^58^ were at serious risk of bias, 1 study had no information,^44^ and another study^18^ was at moderate risk. All studies that performed direct comparison between HD, PD, KT, and dialysis were at moderate risk of bias for fracture.^2^, ^9^, ^19^, ^62^, ^63^ The risk of bias in studies comparing the risk of fracture in HD versus general population was moderate in 1 study,^60^ serious in 1 study,^61^ and critical in 2 studies.^11^, ^59^ The risk of bias in studies comparing the risk of fracture in dialysis to that in the general population was moderate in 2 studies^8^, ^14^ and critical in the other study.^42^ The risk of bias in studies comparing the risk of fracture in KT population with that in the general population was moderate in 2 studies^37^, ^39^ and serious in 1 study.^38^

## Discussion

In this systematic review, we identified 47 studies reporting the risk of fracture in dialysis and KT populations. The incidence of hip fracture in HD group was consistently higher than that reported in PD or KT groups. For overall fracture risk, the incidence seems to be higher in KT compared with HD when considering only studies conducted in cohorts or clinical registries, whereas the incidence is similar in both groups using administrative database studies. Most of these studies have focused on hip or overall fracture, whereas vertebral fracture was rarely addressed. In contrast to fracture incidence, the prevalence of vertebral or overall fracture seems to be similar between HD and KT population. Globally, the risk of bias in these studies was considered critical. The results reinforce the importance of bone fragility as a major health issue in CKD population. Because no direct comparison has been performed due to heterogeneity between studies, the risk of fracture between dialysis and KT population should be further studied.

Dialysis patients (HD and PD) are mostly aged population who suffer from hypogonadism and multiple comorbidities such as diabetes, inactivity, frailty, and cardiovascular disease that predispose them to increased risk of fall and fracture. In addition, some specific factors related to mineral abnormalities in dialysis may further explain the increased risk of fracture in these patients. These include low vitamin D levels, secondary hyperparathyroidism, abnormal calcium metabolism, chronic acidosis state, and higher exposition to heparin due to chronic HD that contribute to low bone mass and worsening of bone microarchitecture and quality.^6^, ^65^, ^66^, ^67^, ^68^ As a matter of fact, bone microarchitecture defects seem to be different between dialysis population as Pelletier and colleagues^69^ have recently shown that trabecular volumetric bone mineral density at the tibia was significantly lower in HD patients compared with PD patients. Nickolas and colleagues^70^ also reported that patients on HD had more severe decreases in cortical bone mineral density and greater increases in cortical porosity at the radius comparatively to PD patients. These higher cortical deteriorations could be explained by a higher level of parathyroid hormone in HD patients. Indeed, it has been suggested that PD patients had lower levels of bone markers, which may protect them from secondary hyperparathyroidism‐induced high bone turnover disease.^71^, ^72^, ^73^ Recognizing the increased fracture risk in CKD population and its determinants especially in subgroups of dialysis patients are therefore of utmost importance as this condition is currently not correctly addressed by the nephrology community.

In KT patients, the increased risk of fracture is mostly explained by the high steroid doses that are used to reduce graft rejection risk in addition to the standard immunosuppressive regimens that are known to affect bone metabolism.^19^, ^74^, ^75^, ^76^, ^77^ After transplantation, a high proportion of patients will continue to have abnormalities in parathyroid hormone levels that will affect bone structure.^78^, ^79^ Indeed, it has been reported that loss of trabecular bone that contributes to reduced bone strength was most severe in patients with both low and high parathyroid hormone levels.^78^, ^79^ Furthermore, KT patients have already a preexistent bone disease that predisposes them to an increased fracture risk post‐transplant. The optimal treatment of bone fragility in KT population remains currently unclear. As the steroid doses given to those patients are progressively lowered after KT, it has been suggested that the risk of hip fracture may be higher in dialysis versus KT patients’ years after KT.^19^ The use of early corticosteroid withdrawal protocol^31^ seems to have a role in preservation of bone mineral density at the central skeleton.^78^ However, it has also been associated with progressive declines in cortical and trabecular bone density at the peripheral skeleton.^78^ At this time, the exact mechanisms leading to bone loss after KT is still not well understood as well as the optimal therapy that should be proposed to these patients in order to reduce fracture risk.

In this systematic review, we have found 5 studies that compared fracture risk between subgroups of dialysis and KT patients. Three studies that compared the risk of hip fracture between HD and PD patients reported a higher risk in HD versus PD, whereas 1 study^19^ observed a higher risk of hip fracture for KT versus combined dialysis patients.^9^ Only 1 study has compared the risk of hip fracture between HD, PD, and KT, which did not reveal a statistical difference. However, the later results should be interpreted with caution, as only 1 subject experienced an episode of fracture in the PD group. Until now, because of lack of adequate studies, the comparative risk of fracture between dialysis and KT population remains therefore poorly understood. Comparative studies on fracture risk and its consequences in advanced CKD population are needed to guide prognostication, to clarify the fracture burden on the health cost, and to help define the design of future prevention trials. Recognizing the subgroups of dialysis or KT patients with the higher risk of fracture will guide the evaluation, planning, and implementation of specific strategies to prevent or treat bone fragility, as well as the organization of care of these aging and already vulnerable patients. In line with the recent KDIGO guidelines in CKD‐MBD,^80^ we believe that it is now time to better target fracture prevention in advanced CKD population to improve the global patients’ quality of life and reduce health cost associated with these severe complications.

Our review has several strengths. We have already registered and published our protocol. We used robust methodology according to the highest standards suggested by Cochrane handbook. We included in our review both dialysis and kidney transplant populations, which have commonly been evaluated separately in previous studies. Our review gives an update on comparative risk of fracture in subgroups of dialysis and kidney transplant patients, who are a highly morbid and vulnerable population not yet adequately addressed in osteoporosis studies. Because the diagnosis of fracture may differ from a study to another, we have reported in this systematic review the results according to the methods used for fracture assessment (administrative data, X‐rays, questionnaire, clinical registry). We believe that this constitutes a strength of our study. Our review has also limitations. It was not possible to calculate pooled data estimates because of lack of adequate studies assessing the same outcome and to heterogeneity among included studies. Therefore, we could not take into account the effect of age on fracture incidence because a meta‐analysis was not performed. Moreover, the context of the fracture was frequently unknown (traumatic or not).^33^ Finally, the assessment of mortality, length of stay, and cost post fracture was limited because we are likely to miss studies conceived specifically to evaluate the association between HD, PD, or KT and these outcomes. However, we believe that studies conducted for these outcomes are poorly available in the literature.

In conclusion, from this review, the comparison of fracture risk in dialysis and kidney transplant population suggests clinically important differences across these groups. Unfortunately, these comparisons were rarely performed and heterogeneity prevented us from conducting a quantitative evaluation of differences. Characterization of fracture risk as well as the societal implications of this complication in dialysis and KT population should clearly be the focus of future studies.

## Disclosures

All authors state that they have no conflicts of interest.

## Supporting information

Supporting Data S1.

## References

[1] U.S. Renal Data System. Morbidity and mortality in patients with chronic kidney disease. USRDS 2011 annual data report. Bethesda, MD: National Institutes of Health, National Institute of Diabetes and Digestive and Kidney Diseases; 2011. p. 68.

[2] Chen YJ , Kung PT , Wang YH , et al. Greater risk of hip fracture in hemodialysis than in peritoneal dialysis. Osteoporos Int. 2014;25(5):1513–8. 24557014 10.1007/s00198-014-2632-6

[3] Pippias M , Stel VS , Abad Diez JM, et al. Renal replacement therapy in Europe: a summary of the 2012 ERA‐EDTA Registry Annual Report. Clin Kidney J. 2015;8(3):248–61. 26034584 10.1093/ckj/sfv014 PMC4440462

[4] CORR. Quick stats, renal replacement therapy for end‐stage kidney disease. Canadian Institute for Health Information (CIHI); 2017 Available at: https://www.cihi.ca/en/corr‐annual‐statistics‐2017.

[5] Tsukamoto Y. [CKD‐MBD (chronic kidney disease‐mineral and bone disorder). KDIGO CKD‐MBD clinical practice guideline]. Clin Calcium. 2010;20(7):1021–7. 20585180

[6] Mac Way F , Lessard M , Lafage‐Proust MH. Pathophysiology of chronic kidney disease‐mineral and bone disorder. Joint Bone Spine. 2012;79(6):544–9. 23177912 10.1016/j.jbspin.2012.09.014

[7] Chen W , Bushinsky DA. Chronic kidney disease: KDIGO CKD‐MBD guideline update: evolution in the face of uncertainty. Nat Rev Nephrol. 2017;13(10):600–2. 28824172 10.1038/nrneph.2017.118

[8] Coco MR , Rush H. Increased incidence of hip fractures in dialysis patients with low serum parathyroid hormone. Am J Kidney Dis. 2000;36(6):1115–21. 11096034 10.1053/ajkd.2000.19812

[9] Stehman‐Breen CO , Sherrard DJ , Alem AM , et al. Risk factors for hip fracture among patients with end‐stage renal disease. Kidney Int. 2000;58(5):2200–5. 11044242 10.1111/j.1523-1755.2000.00394.x

[10] Blayney MJ , Tentori F. Trends and consequences of mineral bone disorder in haemodialysis patients: lessons from The Dialysis Outcomes and Practice Patterns Study (DOPPS). J Renal Care. 2009;35 Suppl 1:7–13. 10.1111/j.1755-6686.2009.00048.x 19222725

[11] Rodríguez‐García M , Gomez‐Alonso C , Naves‐Díaz M , et al. Vascular calcifications, vertebral fractures and mortality in haemodialysis patients. Nephrol Dial Transplant. 2009;24(1):239–46. 18725376 10.1093/ndt/gfn466 PMC2639312

[12] Mirza MA , Karlsson MK , Mellstrom D , et al. Serum fibroblast growth factor‐23 (FGF‐23) and fracture risk in elderly men. J Bone Miner Res. 2011;26(4):857–64. 20928885 10.1002/jbmr.263

[13] Levey AS , Stevens LA , Schmid CH , et al. A new equation to estimate glomerular filtration rate. Ann Intern Med. 2009;150(9):604–12. 19414839 10.7326/0003-4819-150-9-200905050-00006 PMC2763564

[14] Alem AM , Sherrard DJ , Gillen DL , et al. Increased risk of hip fracture among patients with end‐stage renal disease. Kidney Int. 2000;58(1):396–9. 10886587 10.1046/j.1523-1755.2000.00178.x

[15] Mittalhenkle A , Gillen DL , Stehman‐Breen CO. Increased risk of mortality associated with hip fracture in the dialysis population. Am J Kidney Dis. 2004;44(4):672–9. 15384018

[16] Ensrud KE , Lui LY , Taylor BC , et al. Renal function and risk of hip and vertebral fractures in older women. Arch Int Med. 2007;167(2):133–9. 17242313 10.1001/archinte.167.2.133

[17] Beaubrun AC , Kilpatrick RD , Freburger JK , Bradbury BD , Wang L , Brookhart MA. Temporal trends in fracture rates and postdischarge outcomes among hemodialysis patients. J Am Soc Nephrol. 2013;24(9):1461–9. 23744885 10.1681/ASN.2012090916 PMC3752946

[18] Nair SS , Lenihan CR , Montez‐Rath ME , Lowenberg DW , Chertow GM , Winkelmayer WC. Temporal trends in the incidence, treatment and outcomes of hip fracture after first kidney transplantation in the United States. Am J Transplant. 2014;14(4):943–51. 24712332 10.1111/ajt.12652 PMC4117735

[19] Ball AM , GillenDL, Sherrard D , et al. Risk of hip fracture among dialysis and renal transplant recipients. JAMA. 2002;288(23):3014–8. 12479766 10.1001/jama.288.23.3014

[20] Sidibe A , Moore L , Jean S , Mac‐Way F. Fracture risk in dialysis and kidney transplanted patients: a protocol for systematic review and meta‐analysis. Syst Rev. 2017;6(1):37. 28222798 10.1186/s13643-017-0416-8 PMC5320734

[21] Higgins JPT , Green S , editors. Cochrane Handbook for Systematic Reviews of Interventions, Version 5.1.0. The Cochrane Collaboration; 2011.

[22] Moher D , Liberati A , Tetzlaff J , Altman DG , Group P. Preferred reporting items for systematic reviews and meta‐analyses: the PRISMA statement. Int J Surg. 2010;8(5):336–41. 20171303 10.1016/j.ijsu.2010.02.007

[23] Cochrane Methods Bias Group. The ROBINS‐I tool (risk of bias in non‐randomized studies ‐ of interventions). Cochrane Methods. 2016:1.

[24] Braga Junior JW , Neves RM , Pinheiro MM, et al. Prevalence of low trauma fractures in long‐term kidney transplant patients with preserved renal function. Braz J Med Biol Res. 2006;39(1):137–47. 16400474 10.1590/s0100-879x2006000100016

[25] Durieux SM , Mercadel L , Orcel P , et al. Bone mineral density and fracture prevalence in long‐term kidney graft recipients. Transplantation. 2002;74(4):496–500. 12352908 10.1097/00007890-200208270-00011

[26] Patel S , Kwan JT , McCloskey E , et al. Prevalence and causes of low bone density and fractures in kidney transplant patients. J Bone Miner Res. 2001;16(10):1863–70. 11585351 10.1359/jbmr.2001.16.10.1863

[27] Nam JH , Moon JI , Chung SS , et al. Prevalence and risk factors for vertebral fractures in renal transplants. Transplant Proc. 2000;32(7):1877. 11119980 10.1016/s0041-1345(00)01472-x

[28] Nisbeth U , Lindh E , Ljunghall S , Backman U , Fellstrom B. Increased fracture rate in diabetes mellitus and females after renal transplantation. Transplantation. 1999;67(9):1218–22. 10342312 10.1097/00007890-199905150-00004

[29] Grotz WH , Mundinger FA , Gugel B , Exner V , Kirste G , Schollmeyer PJ. Bone fracture and osteodensitometry with dual energy X‐ray absorptiometry in kidney transplant recipients. Transplantation. 1994;58(8):912–5. 7940734 10.1097/00007890-199410270-00009

[30] Ferro CJ , Arnold J , Bagnall D , Ray D , Sharif A. Fracture risk and mortality post‐kidney transplantation. Clin Transplant. 2015;29(11):1004–12. 26313646 10.1111/ctr.12621

[31] Nikkel LE , Mohan S , Zhang A , et al. Reduced fracture risk with early corticosteroid withdrawal after kidney transplant. Am J Transplant. 2012;12(3):649–59. 22151430 10.1111/j.1600-6143.2011.03872.x PMC4139036

[32] Opelz G , Dohler B. Association of mismatches for HLA‐DR with incidence of posttransplant hip fracture in kidney transplant recipients. Transplantation. 2011;91(1):65–9. 21452411 10.1097/tp.0b013e3181fa94d6

[33] Nikkel LE , Hollenbeak CS , Fox EJ , Uemura T , Ghahramani N. Risk of fractures after renal transplantation in the United States. Transplantation. 2009;87(12):1846–51. 19543063 10.1097/TP.0b013e3181a6bbda

[34] O'Shaughnessy EA , Dahl DC , Smith CL , Kasiske BL. Risk factors for fractures in kidney transplantation. Transplantation. 2002;74(3):362–6. 12177615 10.1097/00007890-200208150-00012

[35] Ramsey‐Goldman R , Dunn JE , Dunlop DD , et al. Increased risk of fracture in patients receiving solid organ transplants. J Bone Miner Res. 1999;14(3):456–63. 10027911 10.1359/jbmr.1999.14.3.456

[36] Elmstedt E , Svahn T. Skeletal complications following renal transplantation. Acta Orthop Scand. 1981;52(3):279–86. 7025559 10.3109/17453678109050104

[37] Naylor KL , Jamal SA , Zou G , et al. Fracture incidence in adult kidney transplant recipients. Am J Transplant. 2015;15. 10.1097/TP.0000000000000808 26154389

[38] Vautour LM , Melton LJ 3rd, Clarke BL , et al. Long‐term fracture risk following renal transplantation: a population‐based study. Osteoporos Int. 2004;15(2):160–7. 14666400 10.1007/s00198-003-1532-y

[39] Abbott KC , Oglesby RJ , Hypolite IO , et al. Hospitalizations for fractures after renal transplantation in the United States. Ann Epidemiol. 2001;11(7):450–7. 11557176 10.1016/s1047-2797(01)00226-5

[40] Nair SS , Mitani AA , Goldstein BA , Chertow GM , Lowenberg DW , Winkelmayer WC. Temporal trends in the incidence, treatment, and outcomes of hip fracture in older patients initiating dialysis in the United States. Clin J Am Soc Nephrol. 2013;8(8):1336–42. 23660182 10.2215/CJN.10901012 PMC3731911

[41] Danese MD , Kim J , Doan QV , Dylan M , Griffiths R , Chertow GM. PTH and the risks for hip, vertebral, and pelvic fractures among patients on dialysis. Am J Kidney Dis. 2006;47(1):149–56. 16377396 10.1053/j.ajkd.2005.09.024

[42] Maravic M , Ostertag A , Torres PU , Cohen‐Solal M. Incidence and risk factors for hip fractures in dialysis patients. Osteoporos Int. 2014;25(1):159–65. 23835863 10.1007/s00198-013-2435-1

[43] Ma MK , Yap DY , Yip TP , Lui SL , Lo WK. Charlson co‐morbidity index and albumin significantly associated with fracture risk in peritoneal dialysis patients. Nephrology. 2013;18(5):365–8. 23600370 10.1111/nep.12056

[44] Šimunović I , Pavlovic D , Kudumija B , Mihaljević D , Lovčić V , Jakić M. Bone fragility fractures in hemodialysis patients: Croatian surveys. Coll Antropol. 2015;39(1):71–4. 26040072

[45] Fusaro M , Tripepi G , Noale M , et al. High prevalence of vertebral fractures assessed by quantitative morphometry in hemodialysis patients, strongly associated with vascular calcifications. Calcif Tissue Int. 2013;93(1):39–47. 23494409 10.1007/s00223-013-9722-x

[46] Mares J , Ohlidalova K , Opatrna S , Ferda J. Determinants of prevalent vertebral fractures and progressive bone loss in long‐term hemodialysis patients. J Bone Miner Metab. 2009;27(2):217–23. 19172222 10.1007/s00774-008-0030-x

[47] Kaneko TM , Foley RN , Gilbertson DT , Collins AJ. Clinical epidemiology of long‐bone fractures in patients receiving hemodialysis. Clin Orthop Relat Res. 2007 (457):188–93. 17195813 10.1097/BLO.0b013e318031465b

[48] Inaba M , Okuno S , Kumeda Y , Yamakawa T , Ishimura E , Nishizawa Y. Increased incidence of vertebral fracture in older female hemodialyzed patients with type 2 diabetes mellitus. Calcif Tissue Int. 2005;76(4):256–60. 15692725 10.1007/s00223-004-0094-0

[49] Ureña P , Bernard‐Poenaru O , Ostertag A , et al. Bone mineral density, biochemical markers and skeletal fractures in haemodialysis patients. Nephrol Dial Transplant. 2003;18(11):2325–31. 14551361 10.1093/ndt/gfg403

[50] Fontaine MA , Albert A , Dubois B , Saint‐Remy A , Rorive G. Fracture and bone mineral density in hemodialysis patients. Clin Nephrol. 2000;54(3):218–26. 11020020

[51] Atsumi K , Kushida K , Yamazaki K , Shimizu S , Ohmura A , Inoue T. Risk factors for vertebral fractures in renal osteodystrophy. Am J Kidney Dis. 1999;33(2):287–93. 10023640 10.1016/s0272-6386(99)70302-1

[52] Mohini R , Dumler F , Rao DS. Skeletal surveys in renal osteodystrophy. ASAIO Trans. 1991;37(4):635–7. 1768501

[53] Jamal SA , Leiter RE , Jassal V , Hamilton CJ , Bauer DC. Impaired muscle strength is associated with fractures in hemodialysis patients. Osteoporos Int. 2006;17(9):1390–7. 16799753 10.1007/s00198-006-0133-y

[54] Wagner J Jhaveri KD , Rosen L , Sunday S , Mathew AT , Fishbane S. Increased bone fractures among elderly United States hemodialysis patients. Nephrol Dial Transplant. 2014;29(1):146–51. 24078333 10.1093/ndt/gft352

[55] Chang NT , Lee YH , Hsu JC , et al. Epidemiological study of orthopedic injuries in hemodialysis patients in Taiwan: a fixed cohort survey, 2004‐2008. Clin Interv Aging. 2013;8:301–8. 23682209 10.2147/CIA.S41132 PMC3653676

[56] Wakasugi M Kazama JJ , Wada A , et al. Regional variation in hip fracture incidence among Japanese hemodialysis patients. Ther Apher Dial. 2014;18(2):162–6. 24720407 10.1111/1744-9987.12074

[57] Lavorato C , Del Amo M , Caputo F , et al. Hip fractures in patients in hemodialysis. Revista Nefrologia Dialisis Trasplante. 2009;29(4):137–44.

[58] Jadoul M , Albert JM , Akiba T , et al. Incidence and risk factors for hip or other bone fractures among hemodialysis patients in the Dialysis Outcomes and Practice Patterns Study. Kidney Int. 2006;70(7):1358–66. 16929251 10.1038/sj.ki.5001754

[59] Tentori F , McCullough K , Kilpatrick RD , et al. High rates of death and hospitalization follow bone fracture among hemodialysis patients. Kidney Int. 2014;85(1):166–73. 23903367 10.1038/ki.2013.279 PMC3910091

[60] Wakasugi M , Kazama JJ , Taniguchi M , et al. Increased risk of hip fracture among Japanese hemodialysis patients. J Bone Miner Metab. 2013;31(3):315–21. 23292163 10.1007/s00774-012-0411-z

[61] Rodríguez García M , Gomez‐Alonso C , Naves Díaz, M, et al. Prevalence of vertebral fractures and aortic calcifications in hemodialysis patients: comparison with a population of the same age and sex. Nefrologia. 2003;23 Suppl 2:106–11. 12778865

[62] Lin ZZ , Wang JJ , Chung CR , et al. Epidemiology and mortality of hip fracture among patients on dialysis: Taiwan National Cohort Study. Bone. 2014;64:235–9. 24780875 10.1016/j.bone.2014.04.017

[63] Mathew AT , Hazzan A , Jhaveri KD , et al. Increasing hip fractures in patients receiving hemodialysis and peritoneal dialysis. Am J Nephrol. 2014;40(5):451–7. 25427771 10.1159/000369039

[64] Nikkel L , Mohan S , Zhang C , et al. Reduced fracture risk after kidney transplant with early corticosteroid withdrawal regimens: an analysis of the United States Renal Data System. J Bone Miner Res. 2011;26.

[65] Stein MS , Packham DK , Ebeling PR , Wark JD , Becker GJ. Prevalence and risk factors for osteopenia in dialysis patients. Am J Kidney Dis. 1996;28(4):515–22. 8840940 10.1016/s0272-6386(96)90461-8

[66] McCarthy JT , Kumar R. Renal osteodystrophy. Endocrinol Metab Clin North Am. 1990;19(1):65–93. 2192869

[67] Mac‐Way F , Azzouz L , Noel C , Lafage‐Proust MH. Osteomalacia induced by vitamin D deficiency in hemodialysis patients: the crucial role of vitamin D correction. J Bone Miner Metab. 2014;32(2):215–9. 23794122 10.1007/s00774-013-0480-7

[68] Elder GJ , Mackun K. 25‐Hydroxyvitamin D deficiency and diabetes predict reduced BMD in patients with chronic kidney disease. J Bone Miner Res. 2006;21(11):1778–84. 17002574 10.1359/jbmr.060803

[69] Pelletier S , Vilayphiou N , Boutroy S , et al. Bone microarchitecture is more severely affected in patients on hemodialysis than in those receiving peritoneal dialysis. Kidney Int. 2012;82(5):581–8. 22718192 10.1038/ki.2012.166

[70] Nickolas TL , Stein EM , Dworakowski E , et al. Rapid cortical bone loss in patients with chronic kidney disease. J Bone Miner Res. 2013;28;18–20. 23456850 10.1002/jbmr.1916 PMC3720694

[71] Kurz P , Tsobanelis T , Roth P , et al. Differences in calcium kinetic pattern between CAPD and HD patients. Clin Nephrol. 1995;44(4):255–61. 8575126

[72] Wei M , Esbaei K , Bargman JM , Oreopoulos DG. Inverse correlation between serum magnesium and parathyroid hormone in peritoneal dialysis patients: a contributing factor to adynamic bone disease? Int Urol Nephrol. 2006;38(2):317–22. 16868704 10.1007/s11255-006-0082-6

[73] Dimkovic NB , Bargman J , Vas S , Oreopoulos DG. Normal or low initial PTH levels are not a predictor of morbidity/mortality in patients undergoing chronic peritoneal dialysis. Perit Dial Int. 2002;22(2):204–10. 11990405

[74] Jeffery JR , Leslie WD , Karpinski ME , Nickerson PW , Rush DN. Prevalence and treatment of decreased bone density in renal transplant recipients: a randomized prospective trial of calcitriol versus alendronate. Transplantation. 2003;76(10):1498–502. 14657693 10.1097/01.TP.0000092523.30277.13

[75] Nishioka S , Sofue T , Inui M , et al. Mineral and bone disorder is temporary in patients treated with early rapid corticosteroid reduction after kidney transplantation: a single‐center experience. Transplant Proc. 2014;46(2):514–20. 24656002 10.1016/j.transproceed.2013.11.153

[76] Westenfeld R , Schlieper G , Woltje M , et al. Impact of sirolimus, tacrolimus and mycophenolate mofetil on osteoclastogenesis—implications for post‐transplantation bone disease. Nephrol Dial Transplant. 2011;26(12):4115–23. 21622987 10.1093/ndt/gfr214

[77] Cunningham J. Pathogenesis and prevention of bone loss in patients who have kidney disease and receive long‐term immunosuppression. J Am Soc Nephrol. 2007;18(1):223–34. 17108315 10.1681/ASN.2006050427

[78] Iyer SP , Nikkel LE , Nishiyama KK , et al. Kidney transplantation with early corticosteroid withdrawal: paradoxical effects at the central and peripheral skeleton. J Am Soc Nephrol. 2014;25(6):1331–41. 24511131 10.1681/ASN.2013080851 PMC4033378

[79] Perrin P , Caillard S , Javier RM , et al. Persistent hyperparathyroidism is a major risk factor for fractures in the five years after kidney transplantation. Am J Transplant. 2013;13(10):2653–63. 24034142 10.1111/ajt.12425

[80] Ketteler M , Block GA , Evenepoel P , et al. Executive summary of the 2017 KDIGO Chronic Kidney Disease‐Mineral and Bone Disorder (CKD‐MBD) guideline update: what's changed and why it matters. Kidney Int. 2017;92(1):26–36. 28646995 10.1016/j.kint.2017.04.006

