# Supplementary material for: Fracture Risk in Dialysis and Kidney Transplanted Patients: A Systematic Review
Source: JBMR Plus. 2018 Jul 5;3(1):45–55. doi: 10.1002/jbm4.10067 (PMC6339558; doi:10.1002/jbm4.10067)
Supplement: Supplementary file 1 — Supporting Data S1. [file JBM4-3-45-s001.docx]

**Title: Fracture risk in dialysis and kidney transplanted patients: a systematic review Authors**

Aboubacar Sidibé Msc^1^, David Auguste Msc^2^, Louis-Charles Desbiens^3^, Catherine Fortier Msc^3^, Yue P. Wang^3^, Sonia Jean PhD^4^, Lynne Moore PhD^5^, Fabrice Mac-Way MD^3^

^1^ Centre de Recherche du CHU de Québec, Hôpital Hôtel-Dieu de Québec, Division of Nephrology, Endocrinology and Nephrology Axis, Faculty of Medicine, Department of Social and Preventive Medicine, Laval University, Quebec Canada

^2^ Centre de Recherche du CHU de Québec, Hôpital Saint-Sacrement, Faculty of Medicine, Department of Social and Preventive Medicine, Laval University, Quebec Canada

^3^Centre de Recherche du CHU de Québec, Hôpital Hôtel-Dieu de Québec, Division of Nephrology, Endocrinology and Nephrology Axis, Faculty and Department of Medicine, Laval University, Quebec Canada

^4^ Institut National de Santé Publique du Québec, Medicine Faculty, Department of social and preventive medicine, Laval University, Quebec Canada

^5^ Centre de Recherche du CHU de Québec, Hôpital de l'Enfant-Jésus, Traumatology Axis, Medicine Faculty, Department of Social and Preventive Medicine, Laval University, Quebec Canada

**Corresponding** **author**:

Dr. Fabrice Mac-Way, MD, FRCPC

Centre de Recherche du CHU de Québec, Hôpital Hôtel-Dieu de Québec

10 McMahon, Québec City (Québec), Université Laval

G1R 2J6 Canada

Tel: (418) 691-5464; Fax: (418) 691-5757

Email: fabrice.mac-way@mail.chuq.qc.ca

| **Terms of PICOS/ search steps** | **Terms and synonyms** |
| --- | --- |
| **Intervention**  (Renal Replacement Therapies)   - Kidney transplantation - Hemodialysis - Peritoneal dialysis | 1. “Renal Replacement Therapy” [mesh] or   ((“Renal replacement” [tiab] or “Kidney replacement” [tiab]) and (“therapy” [tiab] or “therapies” [tiab])) or  ((“Kidney” [tiab] or “Renal” [tiab]) and (transplantation*[tiab] or “dialysis” [tiab] or “dialyses” [tiab] or graft*[tiab])) or  “hemodialysis” [tiab] or “hemodialyses” [tiab] or  “Peritoneal Dialysis” [tiab] or “Peritoneal Dialyses” [tiab] or  “Extracorporeal Dialyses” [tiab] or “Extracorporeal Dialysis” [tiab] or  “hemodiafiltration” [tiab |
| **Outcomes**  (fractures) | 1. “Fractures, Bone” [mesh] or   ((“Broken” [tiab] or fracture* [tiab]) and (Bone* [tiab] or “Ankle” [tiab] or “Malleolus” [tiab] or “Trimalleolar” [tiab] or “Bimalleolar” [tiab] or “Femoral” [tiab] or “Hip” [tiab] or “Subtrochanteric” [tiab] or “Trochanteric” [tiab] or “Intertrochanteric” [tiab] or “Femur Neck” [tiab] or “Spontaneous” [tiab] or Pathologic* [tiab] or  “Humeral” [tiab] or “Intra Articular” [tiab] or “Intraarticular” [tiab] or “Radius” [tiab] or “Rib” [tiab] or “Shoulder” [tiab] or “Spinal” [tiab] or “vertebral” [tiab] or  “Tibial” [tiab] or “Ulna” [tiab])) |
| **Combination** | 1. #1 AND #2 |
| **Extraction of animal studies** | 1. “Animal” [mesh] not “human” [mesh] |
| **Exclusion of animal studies** | #3 NOT #4 |

Table S1. Search strategy for Pubmed/Medline

Table S2. Prevalence of fracture in Hemodialysis and Kidney transplant patients

| **First author, publication year** | **Sample** | **Duration of study (Year)** | **Any fracture** | **Hip** | **Vertebral** | **Rib and sternum** | **Foot** | **Other fracture** | **Femur** |
| --- | --- | --- | --- | --- | --- | --- | --- | --- | --- |
| ***Prevalence in Hemodialysis (%)*** | | | | | | | | | |
| *Simunovic 2015* | 767 | 1 | 4.0 |  |  |  |  |  |  |
| *Fusaro 2013* | 387 | 2 |  |  | 55.3 |  |  |  |  |
| *Rodrıguez- Garcıa 2009* | 193 | 2 | 40.0 |  | 26.5 |  |  | 4.2 |  |
| *Mares 2009* | 72 | 1 |  |  | 20.8 |  |  |  |  |
| *Jamal 2006* | 52 | NR | 38.5 |  |  |  |  |  |  |
| *Inaba 2005* | 124 | NR | 18.4 |  |  |  |  |  |  |
| *Urena 2003* | 70 | NR | 30.0 |  | 7.2 | 10.0 |  |  |  |
| *Rodriguez-Garcia 2003* | 99 | NR |  |  | 19.1* |  |  |  |  |
| *Atsumi 1999* | 187 | NR |  |  | 20.9 |  |  |  |  |
| *Fontaine 1999* | 88 | NR | 12.5 |  | 2.3 | 5.7 |  |  | 1.2 |
| *Mohini, Dumler and Rao 1991* | 66 | NR |  |  | 8.0 |  |  |  |  |
| ***Prevalence in Kidney transplant (%)*** | | | | | | | | | |
| *Braga 2006* | 191 | NR | 24.1 |  | 15.2 |  |  |  |  |
| *Durieux 2002* | 59 | 1 | 44.1 |  | 28.8 |  |  |  |  |
| *Patel 2001* | 165 | NR |  |  | 9.1 |  |  |  |  |
| *Nisbeth 1999* | 193 | 23 | 17.0 | 4.2 | 2.6 | 0.5 | 4.7 |  |  |
| *Nam 2000* | 166 | NR |  |  | 38.6 |  |  |  |  |

** Adjusted for age; NR: Not reported*

| **Study author, Country and Year** | **Intervention Group (Incidence or Prevalence)** | **General Population (Incidence or Prevalence)** | **Relative Risk** | **Significance of statistical test** | **Risk of Bias** | **Type of fracture** |
| --- | --- | --- | --- | --- | --- | --- |
| ***Incidence for 1000 person-years, Kidney transplantation*** | | | | | | |
| *Naylor 2015, Canada* | 12.3 | NR | 0.3 * | S | Moderate | Non-vertebral |
| *Vautour 2014, United States* | 128 | NR | 4.8 # | S | Serious | Overall |
| *Abbot 2001, United States* | 0.7 | 0.4 | 4.6 | S | Moderate | Overall |
|  | ***Incidence for 1000 person-years*, Dialysis** | | | |  |  |
| *Maravic, 2013, France* | 1.2 | 0.3 | NR | S | Critical | Hip |
| *Coco 2000, United States* | 13.9 | NR | 17.4║ | S | Moderate | Hip |
| ***Incidence per 1,000 person-years, Hemodialysis*** | | | | | | |
| *Alem 2000, United States* | 7.5 | 5.0 | 4.4 †m | S | Moderate |  |
|  | 13.6 | 7.4 | 4.4 †f | S | Moderate | Hip |
| *Tentori 2000, Multicenter* | 23.0 | NR | NR | S | Critical | Overall |
| *Wakasugi 2013, Japan* | 7.6 | 0.51 | 6.2 †m | S | Moderate | Hip |
|  | 17.4 | 1.8 | 4.9 †f | S | Moderate | Hip |
| ***Prevalence (%), Hemodialysis*** | | | | | | |
| *Rodriguez-Garcia 2009, Spain* | 26.5 | 24.1 | NR | NS | Critical | Vertebral |
| *Rodriguez-Garcia 2003 Spain* | 19.1 | 24.1 | NR | NS | Serious | Vertebral |

Table S3. Comparison of fracture risk between each Modality of renal replacement therapy and general population

*NR: Not reported; NI: No information; KT: Kidney Transplantation; HD: Hemodialysis; PD: Peritoneal Dialysis; S: Significant (p value <0.05 or Confidence Interval excluding the null value); NS: Not Significant (p value ≥ 0.05 or Confidence Interval including the null value 1);* **: Matched on age (±1 year), sex, and index date (±1 year) and adjusting for diabetes, KT as reference group; #: Standardized for age and sex; †: Standardized for Age; ║: Standardized for age, race and sex; m: male ; f: female*

Table S4. Outcomes post-fracture in Dialysis and Kidney Transplantation

| **First author and Year** | **Type of fracture** | **RRT Group** | **Comparator Group** | | **RR** | **Significance** |
| --- | --- | --- | --- | --- | --- | --- |
| **Mortality post-Fracture**  *Dialysis vs General Population* | | | | | | |
| *Coco 2000* | Hip | NR | NR | 2.4 | | S |
| *Maravic 2014* | Hip | 12 *m | 5.5 *m | NR | | S |
|  |  | 7.7 *f | 2.8 *f | NR | | S |
| *Danese 2006* | Overall | 580 # | 217 # | 2.7† | | S |
| *Dialysis with no comparator* | | | | | | |
| *Nair 2013* | Hip | 17.4 * | NA | NA | |  |
| *Zhe-Zhong 2014* | Hip | 3.2 * | NA | NA | | NA |
| *HD vs General Population* | | | | | | |
| *Kaneko 2007* | Overall | 522.6 # | 215.4 # | 2.4 | | S |
| *Rodriguez-Garcia 2009* | Vertebral | NR  500 √# | NR  NR | 4.8 ^║^ | | S |
| *Tentori 2014* | Overall |  |  | 3.7 | | NA |
| *KT vs no comparator* | | | | | | |
| *Nair 2014* | Hip | 2.2 £ | NA | NA | | NA |
| *Ferro 2015* | Overall | 20.7 £ | NA | NA | | NA |
| *Ferro 2015* |  | 32.9 £ | NA | NA | | NA |
| **Hospitalization stays post-fracture (mean ±SD in days)** | | | | | | |
| *Dialysis vs non-dialysis* | | | | | | |
| *Maravic 2014* | Hip | 18 ± 15 m | 13 ± 8 m | NR | | S |
|  |  | 17 ± 12 f | 12 ± 7 f | NR | | S |
| **Hospitalization length or cost due to fracture (Mean ±SD in Euro)** | | | | | | |
| *Dialysis vs non-dialysis* | | | | | | |
| *Maravic 2014* | Hip | 8, 934 ±  5, 420 m | 7, 065 ±  2, 585 m | NR | | S |
|  |  | 8, 443 ±  3, 333 f | 6, 852 ±  1, 735 f | NR | | S |

**: expressed in %; #: per 1000 person-years; †: Overall US dialysis population randomly selected as comparator and relative risk adjusted for age and sex; ║: Adjusted for age and time on HD; √ Exceeded 500 per 1000 person-years; £: Per 100 events; NR: Not reported; NA: Not applicable; S: Significant (p value <0.05 or Confidence interval without the null value);*

*HD: Hemodialysis; KT: Kidney Transplant*

Figure S1. Comparison of hip fracture risk in Hemodialysis, Peritoneal Dialysis and Kidney Transplant population


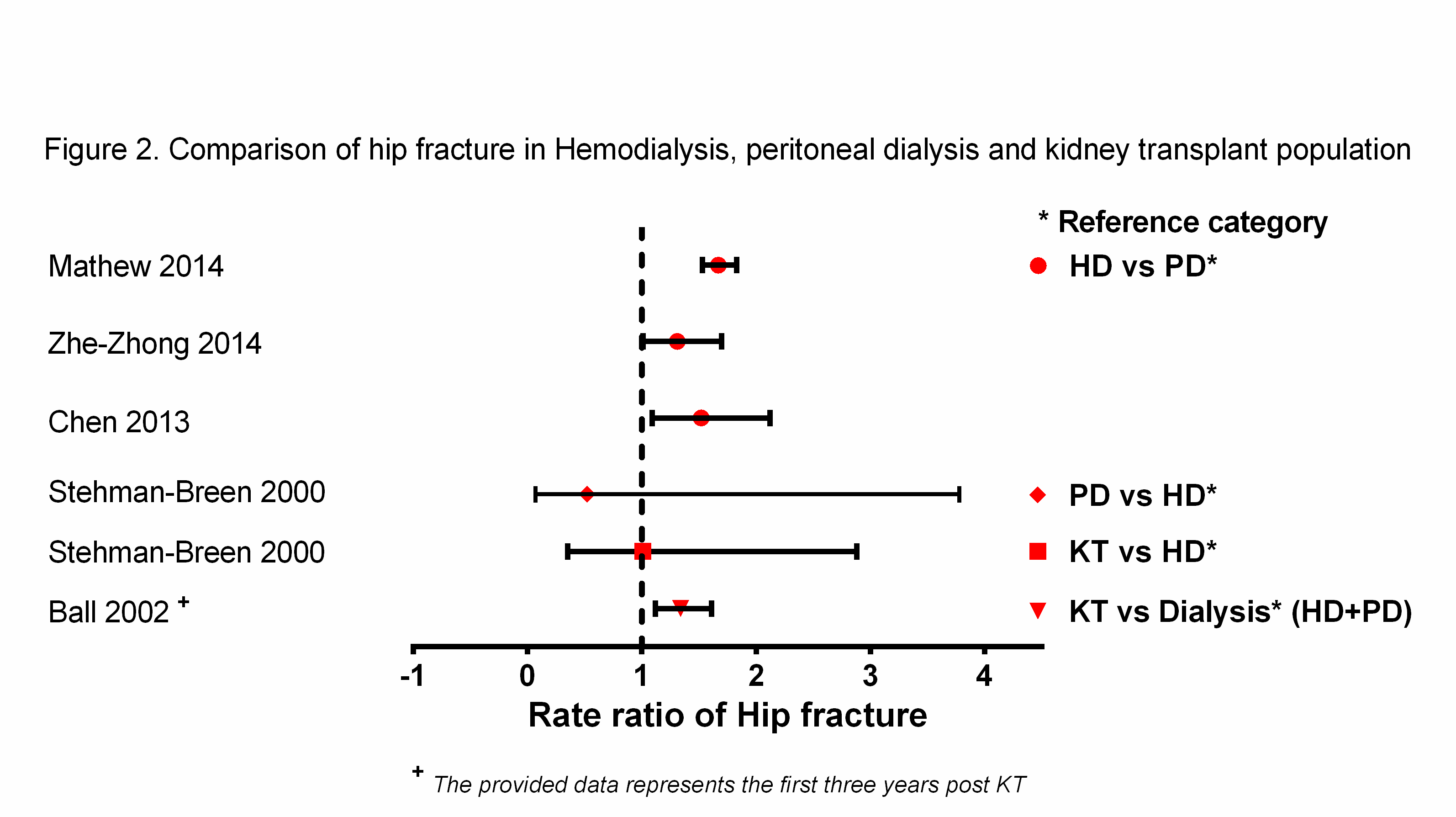


*HD: Hemodialysis; PD: Peritoneal dialysis; KT: Kidney transplantation*
